# Malaria & mRNA Vaccines: A Possible Salvation from One of the Most Relevant Infectious Diseases of the Global South

**DOI:** 10.1007/s11686-023-00712-y

**Published:** 2023-10-12

**Authors:** Yannick Borkens

**Affiliations:** 1 Charité, Charitéplatz 1, 10117 Berlin, Germany; 2 Humboldt-Universität zu Berlin, Unter den Linden 6, 10117 Berlin, Germany

**Keywords:** Global South, Malaria, mRNA vaccinations, Parasitic infectious diseases, Tropical diseases

## Abstract

Malaria is one of the most dangerous infectious diseases in the world. It occurs in tropical and subtropical regions and affects about 40% of the world´s population. In endemic regions, an estimated 200 million people contract malaria each year. Three-quarters of all global deaths (about 600 per year) are children under 5 years of age. Thus, malaria is one of the most relevant tropical and also childhood diseases in the world. Thanks to various public health measures such as vector control through mosquito nets or the targeted use of insecticides as well as the use of antimalarial prophylaxis drugs, the incidence has already been successfully reduced in recent years. However, to reduce the risk of malaria and to protect children effectively, further measures are necessary. An important part of these measures is an effective vaccination against malaria. However, the history of research shows that the development of an effective malaria vaccine is not an easy undertaking and is associated with some complications. Research into possible vaccines began as early as the 1960s. However, the results achieved were rather sobering and the various vaccines fell short of their expectations. It was not until 2015 that the vaccine RTS,S/AS01 received a positive evaluation from the European Medicines Agency. Since then, the vaccine has been tested in Africa. However, with the COVID-19 pandemic, there are new developments in vaccine research that could also benefit malaria research. These include, among others, the so-called mRNA vaccines. Already in the early 1990s, an immune response triggered by an mRNA vaccine was described for the first time. Since then, mRNA vaccines have been researched and discussed for possible prophylaxis. However, it was not until the COVID-19 pandemic that these vaccines experienced a veritable progress. mRNA vaccines against SARS-CoV-2 were rapidly developed and achieved high efficacy in studies. Based on this success, it is not surprising that companies are also focusing on other diseases and pathogens. Besides viral diseases, such as influenza or AIDS, malaria is high on this list. Many pharmaceutical companies (including the German companies BioNTech and CureVac) have already confirmed that they are researching mRNA vaccines against malaria. However, this is not an easy task. The aim of this article is to describe and discuss possible antigens that could be considered for mRNA vaccination. However, this topic is currently still very speculative.

## Introduction

**Fig. 1:**
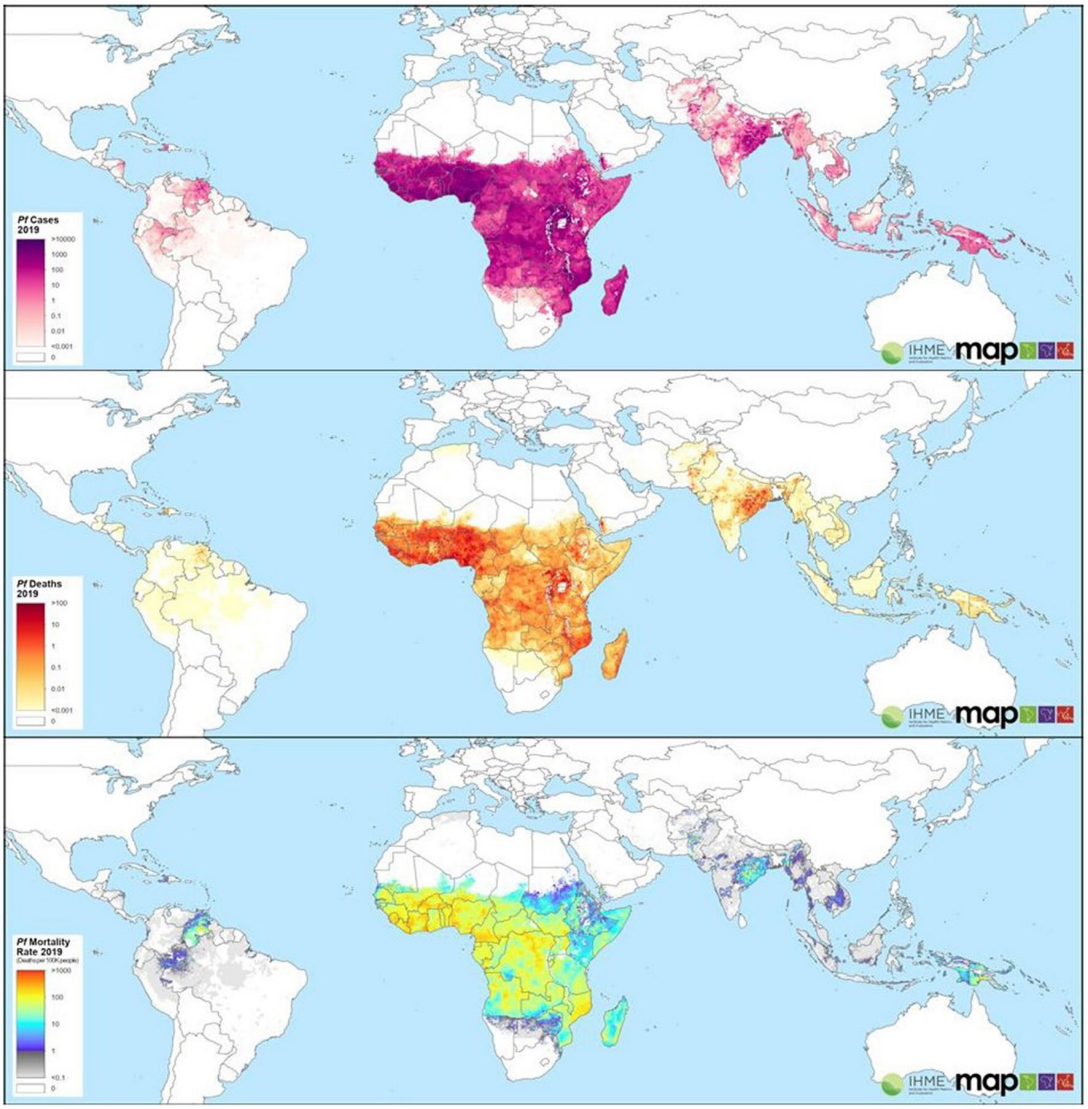
The figure is composed of three different maps from the Malaria Atlas Project. Shown, from top to bottom, are cases, deaths, and mortality rate of *Plasmodium falciparum*. The most recent maps from the MAP are used. These are based on 2020 data. MAP is available under https://malariaatlas.org

Malaria is one of the most relevant infectious diseases in the world [1]. The disease is caused by unicellular parasites of the genus *Plasmodium*. Approximately 40% of the world's population live in areas, where malaria is endemic. It is estimated that approximately 200 million people become ill and 600,000 people die each year. Of these 600,000 deaths, three-quarters are children under the age of 5 (the percentage may vary depending on the source. The World Health Organization estimates 67%). Furthermore, the disease is ancient and closely linked to human history [2]. For example, malaria is believed to be responsible for the deaths of famous historical actors, such as the Pharaoh Tutankhamen, the Visigoth king Alaric, or Pope Innocent VIII [3]. As a tropical infectious disease, malaria primarily affects the populations of tropical countries in the Global South (Africa, Asia and South America). There are often infrastructural and economic problems and challenges in these countries. These, of course, also complicate treatment as well as disease control [4–6]. In countries of the global north, malaria is a relevant travel disease that can occur in returnees from tropical regions. Figure 1 shows the cases, deaths and mortality rate of *Plasmodium falciparum*. The maps were derived from the Malaria Atlas Project, a Collaborating Center in Geospital Disease Modelling of the WHO. The Malaria Atlas Project was launched in 2005 [7–9].

The pathogens that cause malaria are unicellular parasites of the genus *Plasmodium*. The genus is species-rich and infects reptiles, birds as well as mammals. Five species are relevant to humans: *Plasmodium falciparum*, *Plasmodium vivax*, *Plasmodium ovale*, *Plasmodium malariae*, and *Plasmodium knowlesi*. All *Plasmodium* species undergo host switching. They are transmitted by mosquitoes of the genus *Anopheles*, which are also important as definitive hosts. For this reason, the management of the *Anopheles* host are relevant public health measures important for the control of the disease. *Plasmodium* species can be found worldwide.

The genus of the vector *Anopheles* consists of 240 species. Only 40 of these have been shown to transmit malaria [10, 11]. The mosquitoes occur worldwide and can be found in the tropics and subtropics (with the exception of some islands in Oceania, such as New Zealand, Fiji and New Caledonia), as well as in more temperate zones. As the climate warms, reports from colder regions such as Finland are also increasing [12–15]. Since plasmodia depend on the *Anopheles* vector, vector control plays a very important aspect in global malaria management [16, 17]. One of the most successful methods has been the use of mosquito nets, known as bed nets. These were often treated with insecticides in the past [insecticide-treated bed nets (ITNs)] and are still used today [18–20]. Nowadays, however, there is an increasing discussion about the possible harmfulness of these insecticides. Furthermore, own education also plays an important role in the correct application of these methods [21, 22].

When an infected Anopheles mosquito bites a human to suck blood, sporozoites of *Plasmodium* can be transmitted to that human. In humans, the sporozoites first migrate to the liver to infect hepatocytes. In these, they develop into tissue schizonts, which can be up to 80 µm in size. Tissue schizonts contain merozoites that are released into the blood after maturation. Because this initial phase occurs in liver cells, it is referred to as the liver stage, or the pre- or exoerythrocytic phase. This phase is typically asymptomatic. *P*. *ovale* and *P*. *vivax* are able to form so-called hypnozoites. These cells are a dormant form of plasmodia that linger in the hepatocytes (in some cases for up to years) and can later cause a so-called relapse. The signal that triggers this relapse is still unknown [23]. The merozoites released from the liver now infect the erythrocytes. In these, further merozoites are formed over several stages. In this erythrocytic phase, the classic symptoms of malaria, including the fibrous episodes, also occur. These episodes can be traced back to a synchronization of the cell ruptures. In these ruptures, a large number of merozoites are released from many different erythrocytes. In addition to merozoites, gametocytes are also produced in some erythrocytes (female macrogametocytes and male microgametocytes). These gametocytes are later ingested by another *Anopheles* mosquito during the blood meal. Sexual reproduction now takes place in the mosquito (reproduction in humans is asexual). In the intestine of the mosquito, the two gametes fuse to form a zygote. This develops first into a motile ookinete (in the intestine) and finally into an oocyst (in the intestinal wall). In this oocyst new sporozoites are formed, which migrate maturely from the intestine into the salivary glands and are transferred to another human at the next blood meal of the mosquito. Thus, the life cycle is complete. Sporozoite development in the mosquito can take up to 4 weeks, depending on the species and temperature. Neither disease nor symptoms occur in mosquitoes [24, 25].

In the science of parasitology, the different life cycles play a crucial role. Malaria is no exception. Knowledge of life cycles allows the development of different interventions that attack different stages of the life cycle. The mosquito nets described above, which decrease sporozoite transmission by preventing a mosquito bite, are just one example. Chemical methods such as the development of new therapeutics and prophylactics can also be guided by the life cycle, for example, by targeting specific proteins or relevant cell structures. Vaccines are one such example. However, past experience shows that developing of a potent vaccine against malaria is difficult and challenging. In the context of the SARS-CoV-2 pandemic, a new class of vaccines emerged that can and very likely will benefit the development of other vaccines against other pathogens. We are talking about mRNA vaccines, which are based on a messenger RNA that introduces an antigen into the body and thus triggers an immune response. Although mRNA-based vaccines have been the subject of research for some time, the first vaccines were not approved until the 2020s in the context of the SARS-CoV-2 pandemic. The vaccines were developed by various companies, such as BioNTech in Germany, Pfizer or MODERNA, both from the USA. Due to the success of the COVID-19 vaccines, many companies have already announced plans to expand their research to other diseases. In addition to diseases, such as influenza or other coronavirus infections, malaria is high on the agenda here, probably also for marketing reasons. Companies such as BioNTech from Mainz or CureVac from Tübingen have already announced to work on mRNA vaccines against malaria [26, 27]. Countries such as Germany or the USA are also increasingly focusing on vaccines against malaria in their national and global health and development strategies [28, 29]. The question arises as to how realistic such a development is.

## The Rise of mRNA-Vaccination

mRNA was first described in 1961 by Sydney Brenner, François Jacob and Matthew Meselson as a transporter of information relevant to biosynthesis [30]. This discovery likely paved the way for further relevant discoveries and developments, including the development of mRNA-based drugs and vaccines. The first drug based on mRNA is patisiran, which was approved by the EMA (Europe) and FDA (USA) in 2018. Patisiran was developed by the Cambridge-based company Alnylam® Pharmaceutical and is used to treat ATTR amyloidosis, a rare form of amyloidosis. The drug's mechanism of action is based on RNA silencing, in which genes are specifically turned off. Patisiran is marketed under the name Onpattro® [31, 32].

In 1971, there was another milestone relevant to the development of mRNA vaccines. In that year, scientists succeeded for the first time in introducing foreign RNA into frog cells. The introduced RNA was isolated from rabbit reticulocytes. The work demonstrated that cells (in this case, frog cells) or the cell's own translational machinery can also work with RNA derived from other cell types and from completely different species [33]. In 1978, this experiment was successfully repeated with human cells [34]. In addition, in 1978, experiments demonstrated that liposomes serve as possible vehicles for mRNA and can be taken up by cells [35]. In 1989, scientists led by Robert W. Malone were finally able to generate a transfection reagent in which mRNA was introduced into cells using cationic lipids [36]. A year later, RNA generated *in vitro* was successfully introduced into cells. This time without the use of a transfection reagent [37]. Both findings are considered important steps in the development of the first mRNA vaccines.

**Fig. 2:**
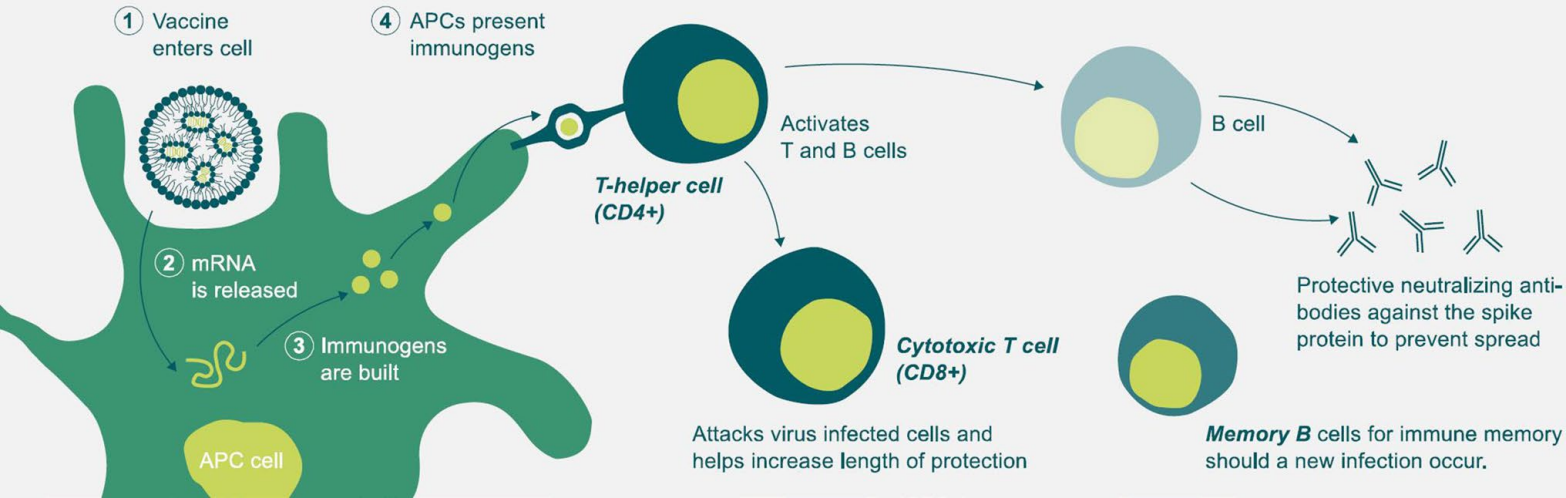
The figure shows the mechanism of how mRNA-based vaccines work. The vaccine encodes the presentation of small and harmless fragments of the COVID-19 virus to immune cells. This enables a rapid and specific immune response upon contact with the actual virus. The figure was derived from BioNTech. The text *mRNA vaccines to address the COVID-19 pandemic* is available under https://biontech.de/covid-19-portal/mrna-vaccines

The first RNA-based vaccines were described in 1993 and 1994. In these years, a cellular immune response was elicited for the first time [38, 39]. In the next year, in 1995, a humoral immune response was elicited as well [39, 40]. Since then, mRNA-based vaccines have been part of academic and pharmaceutical research, with more or less adequate success [41]. Due to these rather successes, the WHO recognized RNA vaccines as a new vaccine class only in 2017 [42]. The final breakthrough of these vaccines is now considered to be the COVID-19 pandemic, beginning in the end of 2019 [43]. The pandemic provided a further acceleration of research and eventually to the first approvals in 2020. Named vaccines are BNT162b2 from Pfizer/BioNTech and mRNA-1273 from Moderna. Both vaccines achieved good results in research [44, 45]. In contrast to inactivated vaccines, in mRNA vaccination only an mRNA encoding a specific vaccine antigen is introduced into the cell. The cell begins to produce the antigen based on the introduced mRNA and presents it to immunoreactive cells. As a result, the immune system responds to the antigen and is thus able to recognize the antigen in the natural pathogen and protect the host. Figure 2 schematically illustrates the mechanism of action. The figure was taken from the official BioNTech website [46]. The antigens used are mainly surface proteins. Already the development of COVID-19 vaccines and the general breakthrough of mRNA vaccines are considered by some to be worthy of a Nobel Prize [47].

## Historical Background of Malaria Vaccines

As described above, the development of a potent malaria vaccine is the goal of many companies and universities. However, the development is not easy and associated with obstacles and difficulties. The development of an effective malaria vaccine is by no means new. As early as the 1960s, malaria was thought to have been conquered. Targeted public health interventions and programs (including programs established during World War II) resulted in a significant drop in cases. However, these rose again significantly at the end of the 1960s. Today, these renewed increases are thought to be due to resistance to DDT and chloroquine developed by *Plasmodium* [48]. DDT, short for dichlorodiphenyltrichloroethane, is an insecticide that has been used since the 1940s. It plays an important role in indoor spraying as well as ITNs. Its advantages include its comparatively simple manufacturing process and its low toxicity to mammals [49]. The WHO has recommended DDT in malaria control since the 1950s. Since 1993, the use of DDT in large-scale projects has been described as inappropriate. The reason for this is the increasing resistance of *Plasmodium*. However, in smaller and more private settings, such as indoor spraying, DDT is still recommended and used [50, 51]. Chloroquine is a drug that has an important role in malaria prophylaxis and therapy. The substance is taken orally as a tablet. Adults are recommended 500 mg per week. It is marketed under the name Resochin. In the past, the drug was often taken in combination with proguanil. The reason for this was also the increasing resistance of *Plasmodium* to chloroquine. However, the efficacy and tolerability of this combination is generally considered to be negative. Nowadays, this combination is no longer commonly used [52, 53].

To counter the problem of increasing resistance, vaccines have been increasingly discussed since the 1960s. Health professionals, but also politicians, hope for a potent malaria vaccine to control and reduce the number of cases of this disease. The wish of a complete eradication, as it was still discussed in the 60s, for example, is nowadays no longer asked by anyone and is considered very unlikely [48]. That vaccines have the potential to regulate or even completely eradicate dangerous diseases is demonstrated by the smallpox vaccination campaign. Smallpox was declared eradicated by the WHO in 1980 thanks to an unprecedented global collaboration [54].

**Fig. 3:**
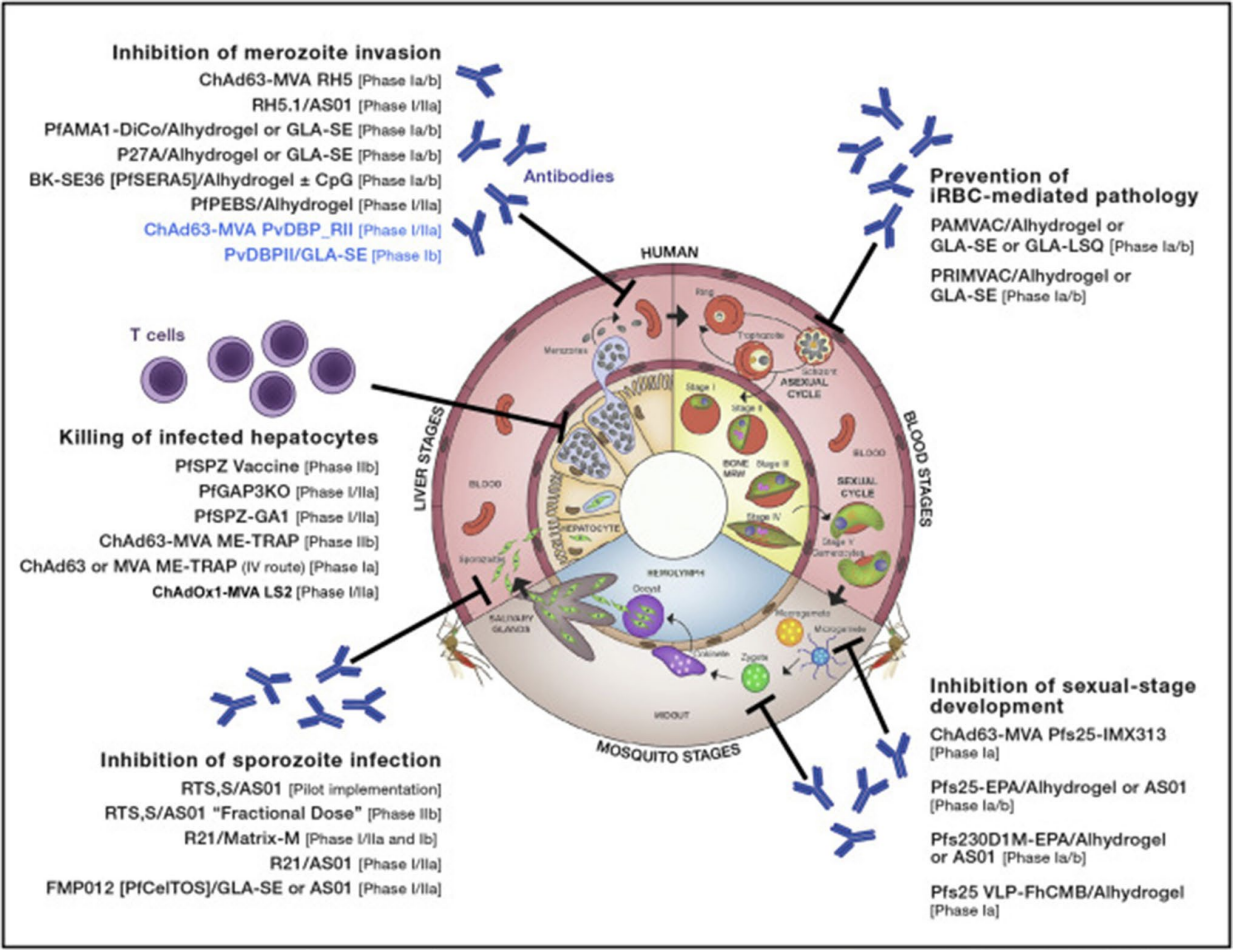
The figure shows the schematic representation of different vaccine platforms against malaria. Shown is not only the mechanism of action, but also the site of action [115]

Today, several vaccine platforms are already available, which differ in their mechanisms of action. Three vaccine classes are distinguished: pre-erythrocyte vaccines, blood-phase vaccines, and transmission blocker vaccines. While pre-erythrocyte vaccines and blood-phase vaccines are primarily intended to elicit an immune response, transmission blocker vaccines are intended to prevent transmission to humans and thus the spread of disease [55]. They act on gametocyte development and target proteins that are important in this development [56]. Pre-erythrocytic vaccines, on the other hand, target the pre-erythrocytic and liver stages of malaria. Effective pre-erythrocytic vaccines are designed to prevent progression of the disease to the blood stage. RTS,S/AS01, a vaccine currently being tested in several trials in Africa, is one such pre-erythrocytic vaccine [55, 57, 58]. Last but not least, blood stage vaccines target the blood phase of infection. These vaccines are believed to provide much more effective protection than pre-erythrocytic vaccines. However, to date, no blood-stage vaccine has reached a phase III clinical trial [55, 59]. Figure 3 shows a schematic representation of the different malaria vaccine platforms.

### Serum *Plasmodium falciparum* Version 66

Serum *Plasmodium falciparum* version 66, also referred to as SPf66, is among the first vaccines developed against malaria. It is based on chemically synthesized 45-amino acid peptides derived from fractions of four different proteins extracted from *Plasmodium falciparum* [60]. The vaccine was developed in Colombia by a scientist, Manuel Patarroyo, who gave his patent to the World Health Organization. He was quickly hailed as a biomedical hero in Colombia [61]. Pilot trials in Africa, however, showed rather little effect. The number of first infections with *P*. *falciparum* was reduced by 28%. However, SPf66 showed no effect on *P*. *vivax*. The same was true for hospitalizations for severe malaria [62]. Furthermore, follow-up studies showed that the incidence of clinical malaria was statistically significantly higher in children receiving SPf66. This effect was also related to dose. It was most pronounced in children who received a high dose (1.0 mg) of SPf66 [63, 64]. Today, SPf66 has no role as a potential vaccine against malaria. Other vaccine platforms show much better effects and immune responses.

### RTS,S/AS01

RTS,S/AS01 is one of the most promising vaccines currently available. Developed by the British company GlaxoSmithKline, RTS,S/AS01, which is marketed under the name Mosquirix^tm^ [65]. It targets the circumsporozoite protein (CSP) secreted by the sporozoite phase of *Plasmodium* and located on its cell surface. In studies, RTS,S/AS01 achieved 30% efficacy against uncomplicated malaria [66]. As of April 2019, the WHO is testing the vaccine in Africa (Malawi, Kenya, and Ghana) [55, 67]. As RTS,S/AS01 is the first vaccine to be tested in a phase 4 trial under real epidemiological conditions, the question arises whether the development of an effective malaria vaccine has now entered the final phase [66]. However, there are also critical issues. For example, efficacy is quite low at 30% [68, 69]. In addition, it is possible that RTS,S/AS01 increases mortality in young girls. This has been demonstrated in studies, but there are different interpretations. For example, the results could also be due to statistical factors (e.g., lower overall mortality in the female control group). Further studies and more research are needed to provide more information [70, 71]. The dosage and booster of RTS,S/AS01 are also currently the subject of research. Studies show that there is a significant increase in severe malaria in older RTS-vaccinated children if they have not received booster vaccination [72, 73]. Dosing is another issue that needs to be clarified through research [74].

In addition to RTS,S/AS01, another vaccine targets the CSP. R21/Matrix-M is a relatively new vaccine being developed by the University of Oxford, among others. In a phase II trial conducted in Burkina Faso, a total of 450 children aged 5–17 months were vaccinated. They received one dose three times at 4-week intervals during the malaria season. A rabies vaccine was used as a control. An efficacy of up to 77% was achieved in the study. This efficacy is higher than other vaccine candidates, whose efficacy is approximately 56% [75–77].

### PfSPZ (PfSPZ-CVac)

PfSPZ is another candidate being developed by Rockville, Maryland-based Sanaria Inc. PfSPZ uses sporozoites that are unable to divide to trigger an immune response in the body. To prevent division, these sporozoites are irradiated with radioactive radiation to weaken them. The name of the vaccine is derived from this process: Pf stands for *Plasmodium falciparum*, SPZ for sporozoite. PfSPZ has already shown good results in small studies [78, 79]. Three doses of 5.12 × 10^4^ PfSPZ prevented infection in 9 of 9 volunteers. Ten weeks after the dose, the volunteers were infected with malaria in a controlled manner [79]. A dose of 5.12 × 10^4^ PfSPZ achieved 100% protection, a dose of 3.2 × 10^3^ achieved 67% protection, and a dose of 1.28 × 10^4^ achieved 33% protection [79]. These results make PfSPZ, also known as PfSPZ-CVac, a promising candidate. It is marketed under the name Sanaria®.

### Genetically Modified Plasmodia in Vaccine Research

Another possibility to develop a robust vaccine against malaria is the use of genetically modified plasmodia. For example, rodents "vaccinated" with uis3-deficient sporozoites showed a robust immune response to them [80]. UIS3 (upregulated infective sporozoites gene 3) is a gene essential for the early liver stage. uis3-deficient sporozoites infect hepatocytes but are unable to cause blood stage infections, making them interesting candidates for vaccination research. In general, the liver stage of *Plasmodium* infections remains one of the best targets for live attenuated vaccines [81, 82].

### Testosterone in Vaccine Research

Testosterone is a crucial steroid hormone. As an important messenger substance, it also interacts with pathogens in the body. It also has a certain influence on plasmodia. For example, in 1991, research showed that host testosterone has a negative effect on the self-healing ability of *Plasmodium chabaudi*. Pretreatment of the host with testosterone reduced the self-healing ability to 60% (1-week pretreatment), 40% (2-week pretreatment) and finally to 0% (3-week pretreatment) [83, 84]. The loss of self-healing correlated with increased expression of five proteins in the spleen cells of mice treated with testosterone for 3 weeks and an increased ability to stimulate the proliferative response to T cells triggered by concanavalin A [83]. However, these results were shown only *in vivo* and not *in vitro*. Therefore, it can be assumed that the influence of testosterone is not direct but indirect, i.e., via a testosterone metabolite or testosterone-induced factors [83, 85].

### The Future of Malaria Vaccines 

A total of about 70 vaccine candidates are currently in various stages of scientific and clinical research. Optimistically, an effective vaccine against malaria is only a matter of time. The novel mRNA vaccines offer a further opportunity to develop effective vaccines. The Tübingen-based company CureVac is already planning the first registration trials [77]. The question remains which antigens are suitable for the development of these vaccines and which are capable of eliciting a robust immune response.

## Potential Targets of mRNA Vaccines Against Malaria

At this point, it is not really possible to say which way future mRNA vaccines will work and which antigens they will target. This is partly because potential candidates are still in the research phase and companies in particular do not publicly discuss these phases. Another problem is with *Plasmodium* and its complex cell structure. Past experience shows that the development of drugs and agents against complex Protozoa is notoriously difficult, time-consuming, and expensive [86, 87]. On the other hand, mRNA vaccines have advantages that do not exist with other platforms. On one hand, they are highly flexible and can thus be quickly adapted to possible mutations [88, 89].

In the following, some possible antigens will be described. It can be assumed that future mRNA vaccines could target on of these antigens. Among other things, because earlier non-mRNA vaccines already target these proteins. However, despite all this, this part of the article is clearly more speculative than the rest of the text and should be read with this knowledge in mind. What can be assumed with a high degree of certainty is that surface proteins will play a relevant role in development. Surface proteins are crucial for pathogen–host interaction. As exposed structures, they are the first pathogen molecules to come into contact with the host molecules and, therefore, with the host immune response [90, 91]. Thus, the mRNA vaccines against SARS-CoV-2 target a spike protein (spike glycoprotein or S-glycoprotein) which is a fusogenic protein located in the membrane of SARS-CoV-2 and interacts with the host receptor ACE2. The circumsporozoite protein of *Plasmodium* is also such a surface protein. Surface proteins (and membranes in general) are major players in evolution [92, 93]. Knowledge about this evolution can also be crucial for medical research. Besides surface proteins, which are located in the outer envelope of cells, cellular compartments are of interest. These compartments are located in eukaryotic cells and are surrounded by at least one membrane. They probably arose by endosymbiosis, which occurred several times during evolution. Some members of the Apicomplexa, including *Plasmodium*, possess such a compartment: the apicoplast.^1^ These are a non-photosynthetically active plastid surrounded by four membranes [94]. It is an important part of various metabolic pathways, such as the fatty acid synthesis, isprenoid precursor synthesis and parts of the heme biosynthesis. As a relevant cell structure, it is a relevant target of various drugs and therapeutics [95]. However, it is questionable how realistic an mRNA vaccine targeting the apicoplast is. Surface proteins are probably the more realistic target. Research revealed that 20–30% of all expressed proteins are housed within the cell membrane. In humans, about a quarter of the proteins encoded by the genome carry at least a stretch of sequences predicted to be transmembrane domains. In addition to infection, surface proteins play important roles in communication, sensing, and energy conservation [96]. A broad spectrum for potential targets of antibodies and vaccines.

### Circumsporozoite Protein

**Fig. 4:**
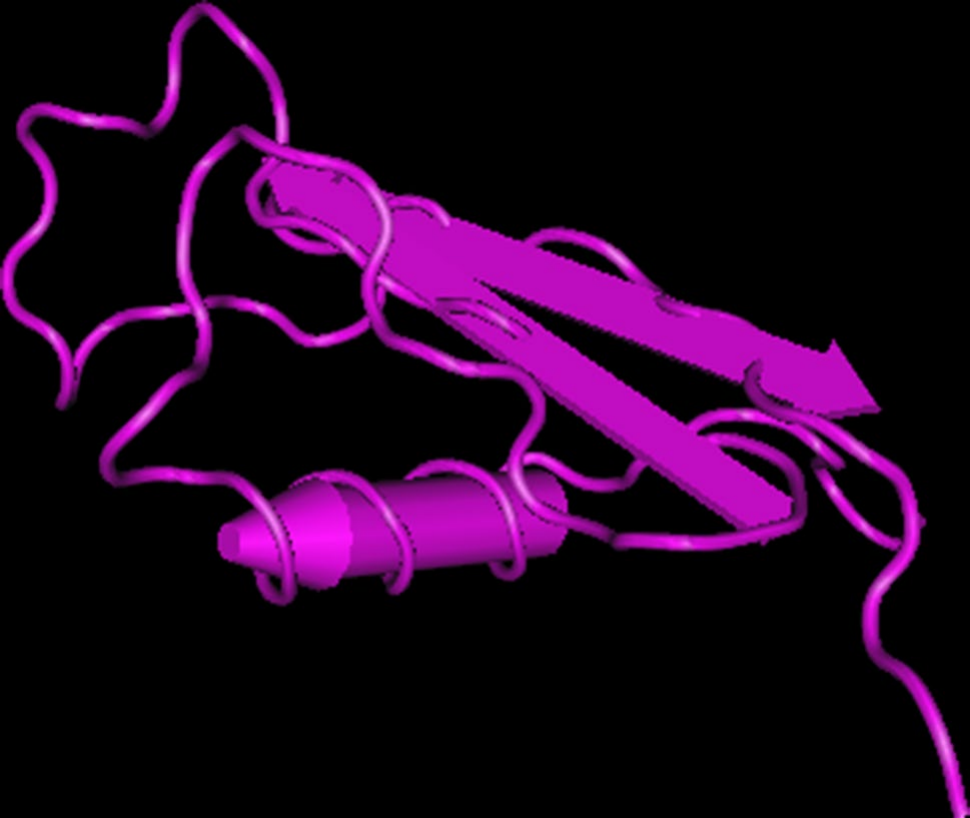
The figure shows the 3D molecular structure of the circumsporozoite protein (CSP) of *Plasmodium falciparum*. CSP is located on the surface of *P*. *falciparum* sporozoites and is important for parasite development, motility, and invasion of host hepatocytes. Therefore, it is an important target for potential vaccines. A well-known example is the RTS,S/AS01 vaccine, an advanced pre-erythrocytic vaccine developed by GlaxoSmithKline and the PATH Malaria Vaccine Initiative [116]

The circumsporozoite protein (CSP) is a surface protein consisting of three regions: the N-terminus, a 4-amino acid repeat region, and the C-terminus. While the N-terminus is capable of binding sulfate proteoglycans, the C-terminus possesses a thrombospondin-like type I repeat domain (TSR) [97]. CSP is formed during the formation of sporozoites in the mosquito. It is important for the migration of the parasite from the mosquito to the intermediate host as well as during infection of hepatocytes [98]. It appears as a highly elongated protein (R(h) 4.2 or 4.58 nm). High resolution microscopy reveals flexible rod-like structures with a ribbon-like appearance [97, 99]. The protein is found in various strains (these infect both humans and non-human primates). For this reason, CSP is considered highly conserved, which in turn suggests that CSP has become conserved by natural selection. This is advantageous for the development of new therapeutics, because mutations in these conserved sequences are rare. In addition, the successes of RTS,S/AS01 indicate that CSP is a good candidate for potential mRNA vaccines [66]. PubMed already lists initial studies showing robust immune responses. For example, researchers demonstrated a functional and protective immune response in mice after injection of CSP mRNA against the pathogen *Plasmodium berghei*, a *Plasmodium* species that infects rodents. A transgenic parasite strain of *P*. *berghei* was used. For the study, sporozoites were injected intravenously at three doses (300, 1000, and 3000 sporozoites). Parasitemia in the blood was monitored for 14 days. A total of three doses of PfCSP mRNA-LNP1 were used in the study. A 30-µg dose, a 10-µg dose, and a dose containing only LNP1 (which served as a control). Animals were vaccinated twice, 3 weeks apart. Two weeks after the last vaccination, blood samples were collected and analyzed. The high dose achieved a vaccination efficacy of 40%, while the lower dose achieved a vaccination efficacy of 20%. The non-vaccinated control group invariably developed blood-stage infection. The results make it a compelling candidate for further investigation and suggest that CSP-based mRNA vaccination is very likely [100]. However, it is questionable whether this vaccination can enter the clinical phase as early as 2022. A 3D model of CSP is shown in Fig. 4.

### Merozoite Surface Protein-1

The merozoite surface protein-1 (MSP-1) is another surface protein that occurs in merozoites. MSPs are synthesized at the beginning of the schizogenesis and important in the infection of red blood cells. Located on the merozoite surface, MSPs are anchored to the membrane with glycophosphatidylinositol, a glycolipid. The MSP-1 complex binds to Spectrin, a surface protein of erythrocytes which is important for cell stability. In addition to MSP-1, there are other MSPs. MSP-2 plays a similar role as MSP-1, although its function has not yet been clarified. The roles of MSPs MSP-3, MSP-6, MSP-7 and MSP-9 are also unclear [101]. Vaccines (as well as drugs) directed against merozoites usually target MSP-1, but these MSPs are not as conserved as, for example, CSP and exhibit sequence variation between the different *Plasmodium* species [102]. This complicates the development of vaccines and therapeutics. The mRNA of MSP-1 is 5466 base pairs long [103]. In addition to MSP-1, MSP-3 is also of interest as a vaccine antigen [104].

### *Plasmodium falciparum* Reticulocyte Binding Homologue 5

In addition to MSPs, Rhs are important for erythrocyte invasion. Rh means reticulocyte-binding homolog. The family includes Rh1, Rh2a, Rh2b, Rh4 and Rh5. Rh5 is the smallest protein of this family. One reason for this is that Rh5 is the only Rh protein that does not have a transmembrane domain. This is unique among Rh proteins [105]. Rh5 reacts with the erythrocyte receptor basigin (BSG) [106, 107]. BSG is a glycoprotein that is involved in carbohydrate metabolism and immune signal transduction, among other functions. In addition, BSG is important as a determinant of the OC blood group system [108]. Studies have shown that both anti-Rh5 monoclonal and polyclonal antibodies are able to block Rh5–BSG interactions [105, 109]. However, Rh5 also presents several problems that complicate the development of new vaccines. Rh5 does not function in isolation, but only as part of a multiprotein complex consisting of the Rh5-interacting protein, the cysteine-rich protein antigen, and P113. This complex greatly facilitates binding to the erythrocyte [105, 110]. In addition, polymorphisms play an important role in vaccine development as barriers. Proteins exposed to the immune system during invasion often exhibit some diversity, probably due to the pressure of the immune system [111]. A good example is AMA1, which has already been tested as an antigen candidate for blood vaccines. Rh5 appears to be more conserved, favoring vaccine development [105].

### Pfs25

Also expressed on the surface is Pfs25. Pfs25 is a sexual stage antigen as it is present on the surface of zygotes and ookinetes. Since antibodies against Pfs25 are capable of completely arresting oocyst development, Pfs25 is of interest for the development of transmission-inhibiting vaccines [112]. Monoclonal antibodies directed against Pfs25 have been shown to prevent transmission from humans to mosquitoes by blocking the transition from ookinete to oocyst [113]. However, no robust antibody titer has yet been established in humans. This is likely due to the poor recognition of Pfs25 by human CD4 + T cells [114]. Assuming that the primary purpose of mRNA vaccination is to protect the individual rather than to prevent transmission, this lack of recognition is a major drawback. Thanks to ongoing research, Pfs25-based vaccines that elicit a robust immune response are likely. However, to provide rapid and effective mRNA vaccines, other proteins are likely to be more suitable.

## Conclusion

mRNA vaccines have been in development for several years. However, it is only the current COVID-19 pandemic that has practically triggered a medical revolution. Working and highly effective vaccines against SARS-CoV-2 were developed in record time. It is, therefore, not surprising that the responsible companies are expanding their focus to other pathogens. In the future, we will have different mRNA vaccines against different diseases. In addition, one of them will probably be against malaria. This perspective highlights which proteins and antigens are candidate for potential mRNA vaccines. Although the circumsporozoite protein is probably one the best candidates, other antigens also have the potential to form the basis for effective mRNA vaccination. However, even these considerations are highly speculative at the moment. What can be said in conclusion is that a potent vaccination against malaria is currently probably only a question of time. The knowledge gained from the SARS-CoV-2 pandemic and the associated development of novel vaccines (including mRNA vaccines) will have a positive impact on future developments. Even if the first approved vaccines against malaria are not mRNA vaccines.

## Data Availability

Not applicable.

## Footnotes

1 The name *Apicomplexa* is derived among others from this Apicoplast.
